# Longitudinal changes in telomere length in PCB-exposed individuals: interaction with CMV infection

**DOI:** 10.1007/s00204-021-03019-x

**Published:** 2021-03-19

**Authors:** Fabian Beier, Andre Esser, Lucia Vankann, Anne Abels, Thomas Schettgen, Thomas Kraus, Tim H. Brümmendorf, Patrick Ziegler

**Affiliations:** 1 Department of Hematology, Oncology, Hemostaseology, and Stem Cell Transplantation, Medical Faculty, RWTH Aachen University, Aachen, Germany; 2 Institute for Occupational, Social and Environmental Medicine, Medical Faculty, RWTH Aachen University, Pauwelsstraße 30, 52074 Aachen, North Rhine Westphalia Germany

## Abstract

We recently demonstrated a significant shortening of age-adapted telomere length (TL) in lymphocytes of polychlorinated biphenyls (PCB)-exposed individuals. Here, we analyzed TL in individuals of the same PCB-exposed cohort during a 6-year follow-up period, investigating the change in TL between the first and second measurement as a function of time, concentration of PCBs and cytomegalovirus (CMV) infection. The age-adjusted TL of lymphocytes within the cohort of PCB-exposed individuals recovered from a first assessment in 2011 to a second assessment in 2017. Remarkably, if the concentration of lower chlorinated PCBs (LC PCBs) in 2011 was high (≥ 0.055 µg/L), the TL of CMV seropositive individuals remained significantly shortened both compared to age-adjusted controls as well as intra individually. This was confirmed by analysis of covariance as well as by multivariate linear mixed effects models. Since telomeres are responsive to various stress response pathways, including viral infection, we conclude that PCBs could contribute to immune senescence-like phenotypes associated with CMV infections and exacerbate negative aspects associated with the aging of the immune system.

**Supplementary Information:**

The online version contains supplementary material available at 10.1007/s00204-021-03019-x.

Telomeres are highly repetitive nucleotide sequences at the end of chromosomes. In normal somatic tissues, telomere length (TL) decreases with aging in vitro and in vivo and therefore reflects the proliferative history of somatic cells (Blasco 2005; Rufer et al. 1999). A few cell types, such as proliferating lymphocytes, counteract replicative telomere shortening through the upregulation of telomerase, an enzyme which elongates telomeres (Hiyama et al. 1995; Flores et al. 2006). In previous experiments we could demonstrate that polychlorinated biphenyls (PCBs) inhibit telomerase gene expression resulting in significant shortening of telomere length (TL) in lymphocytes (Ziegler et al. 2017) (HELPcB cohort, Fig. 1a). PCBs are organic pollutants which can cause considerable damage to human health with chronic toxicity and long-term effects on the immune system even detected at exposures to low levels (Lauby-Secretan et al. 2013; Weisglas-Kuperus et al. 2000). Shorter TL in lymphocytes is associated with a higher incidence of infection and clinical illness (Cohen et al. 2013). Comparing the individual follow-up in the HELPcB cohort from 2011 to 2017, we recognized that shortening of TL in lymphocytes had lost significance compared to age-adjusted controls (Delta-TL: − 0.38 kb, not significant) and showed a tendency towards the age-adjusted median (Fig. 1b). In contrast, the age-adjusted TL in granulocytes from samples of the same cohort did not differ from controls in both measurements. Focusing on PCB levels, no significant difference in the level of higher chlorinated PCBs (mean concentration HC PCB 2011 = 5.12 μg/L vs mean concentration HC PCB 2017 = 5.02 μg/L; *p* = 0.93) nor in the level of dioxine-like PCBs (mean concentration DL PCB 2011 = 1.46 μg/L vs mean concentration DL PCB 2017 = 0.94 μg/L; *p* = 0.11) was observed. In contrast, levels of lower chlorinated PCBs (mean concentration LC PCB 2011 = 0.61 μg/L vs mean concentration LC PCB 2017 = 0.12 μg/L; *p* = 0.01) decreased significantly over time (Fig. 1c). Since PCBs inhibit telomerase gene expression in proliferating lymphocytes (Ziegler et al. 2017; Vasko et al. 2018), we reasoned that telomeres of individuals periodically exposed to a recurrent antigen should be more susceptible to telomere shortening. To proof this hypothesis in the HELPcB cohort, we focused on CMV positive and CMV negative individuals. In line with other studies (van de Berg et al. 2010), TL was found to strongly correlate with age in lymphocytes and telomere attrition was exacerbated in CMV-positive individuals (Fig. 2a). Next, we analyzed the evolution of TL during follow-up from 2011 to 2017 in groups of CMV seropositive and seronegative individuals, taking into account the concentration of LC PCBs determined in 2011 (Fig. 2b). A repeated measures ANCOVA analysis adjusted for smoking habits and daily alcohol intake confirmed that TL recovered from 2011 to 2017 in lower contaminated individuals and was independent of CMV infection (Fig. 2b, black line; PCB ≤ 0.055 µg/L). In higher contaminated individuals, recovery of TL was observed in CMV seronegative individuals, while it was virtually absent in CMV seropositive individuals (Fig. 2b, red line; cut-off for PCB: ≥ 0.055 µg/L). Figure 2b shows exemplary data for PCB 28, similar results were obtained for PCB 101 and PCB 52 as well as the aggregate of LC PCBs, (supplemental table 1). In addition, the interaction between CMV infection and PCB concentration on TL was confirmed using a multivariate linear mixed effects model analysis (supplemental table 2): PCB 28 (*t* = −1.487 | *p* = 0.037) and the sum of the LC PCBs (*t* = − 1.504 | *p* = 0.031) showed a significant negative influence on TL at high concentrations. The strongest influence on TL was exerted by the interaction between a high LC-PCB burden (≥ 0.055 µg/L) and CMV seropositivity. This could be demonstrated for PCB 28 (*t* = − 2.840 | *p* < 0.01), PCB 52 (*t* = − 2.854 | *p* < 0.01), PCB 101 (*t* = − 2.065 | *p* = 0.04) and for the aggregate of the LC PCB (*t* = − 2.808 | *p* < 0.01).

**Fig. 1:**
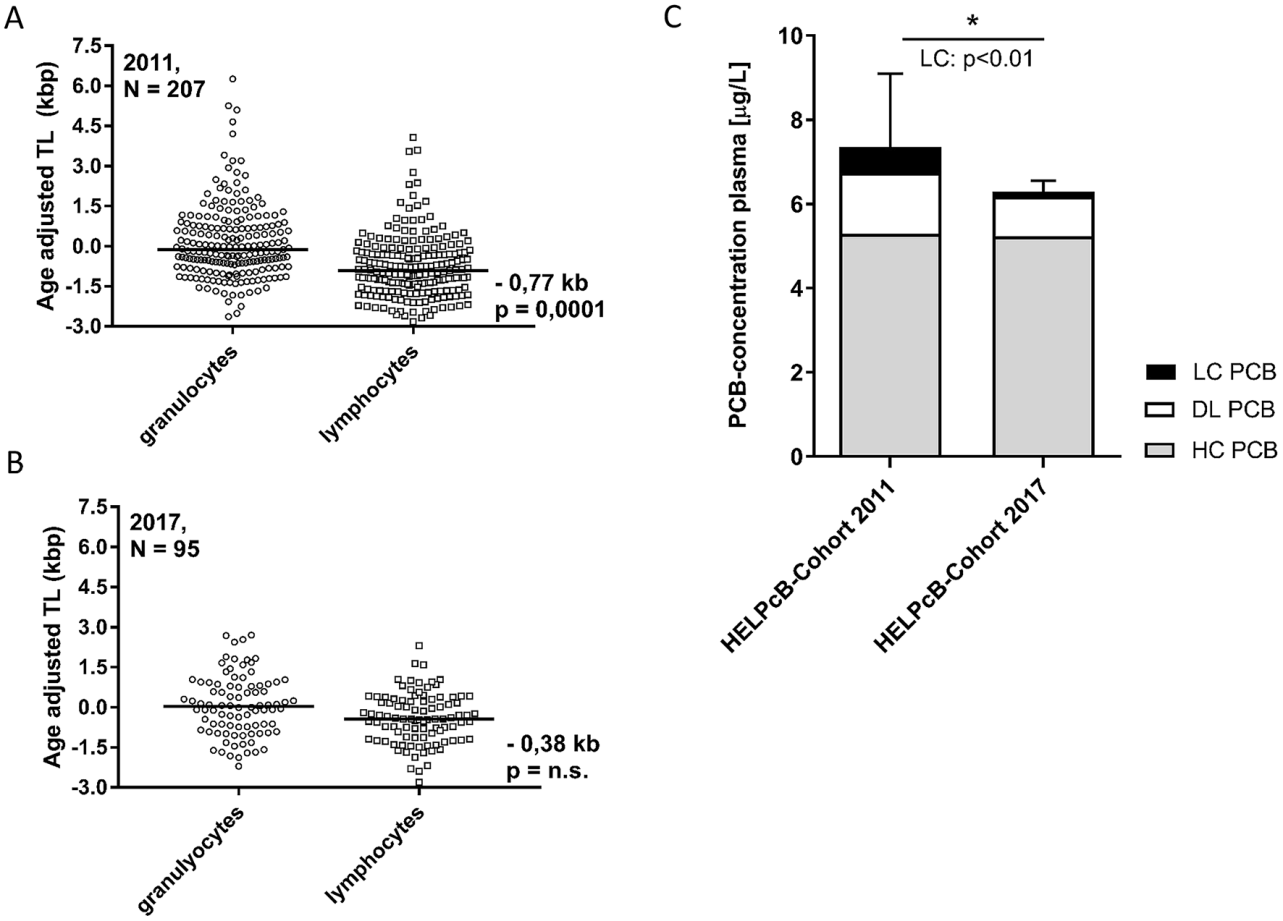
Longitudinal analysis of telomere length in peripheral blood cells of individuals occupationally exposed to PCBs. **a**, **b** Median loss of TL in the peripheral blood of individuals from the HELPcB cohort in 2011 and 2017 measured by flow-FISH. For comparison, telomere length values were age-adjusted using the slope parameter of the age versus telomere length regression of a healthy control group (*N* = 104). **c** Mean plasma levels for Σ higher chlorinated PCBs (gray), Σ dioxin-like PCBs (white) and Σ lower chlorinated PCBs (black) from corresponding plasma samples in 2011 and 2017. Statistical differences are indicated

**Fig. 2:**
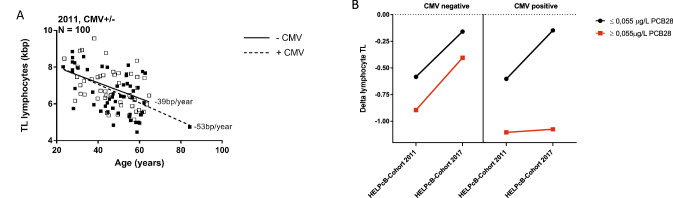
Influence of initial plasma LC-PCB levels on longitudinal TL changes: dependence on CMV seropositivity. **a** Age regression of TL of lymphocytes in dependence of cytomegaly seropositivity. Individuals from the HELPcB cohort were tested for the presence of CMV IgG antibodies in 2015. The results were applied retrospectively to TL data from 2011. **b** Longitudinal development of lymphocyte TL (delta lymphocyte TL) as a function of initial PCB28 plasma levels and CMV seropositivity. Lower and higher concentrated PCB28 levels were split at the median (0.055 µg/L)

In summary we demonstrate, that the age-adjusted TL of lymphocytes within the HELPcB cohort recovered from a first assessment in 2011 to a second assessment in 2017. This correlates with the elimination of LC PCBs from the body and is underpinned by the fact, that the inhibition of *hTERT* expression within plasma samples from the same cohort is relieved over time (Vasko et al. 2018). Decreasing LC-PCB levels would therefore allow T cells to upregulate hTERT expression during clonal proliferation, limiting further telomere attrition and paving the way for TL recovery according to the biological age of the host. In addition our data support the hypothesis, that chronic infections, such as CMV, in combination with a high burden of LC PCB could be responsible for the missing TL recovery in individuals of the HELPcB cohort. The shortening of telomeres increases with age and can be accelerated by natural stressors such as viral infections (CMV, EBV, HIV). A latent CMV infection favors immune senescence and correlates with an accelerated shortening of telomeres in the T-cell pool, a poor antibody response to influenza vaccination and poor immunity to EBV infection (Cicin-Sain et al. 2012). In addition, epidemiological studies with healthy, elderly people (> 65 years), have shown that CMV carriers have an impaired life expectancy as compared to non-infected controls (Wikby et al. 2006). Interestingly, adverse effects on the immune system, which have been described in older people, are also related to PCB exposure. Since in CMV seropositive individuals with a high PCB exposure, age-adapted TL remains significantly shortened over a period of six years, we postulate that PCB mediated accelerated telomere shortening in lymphocytes could thus contribute to the immune senescent phenotype related to CMV infection and thus accelerate negative aspects associated with the aging of the immune system. However, additional longitudinal follow-up is needed to further substantiate the latter hypothesis e.g. by studying whether in this cohort, accelerated TL shortening is correlated with a higher incidence of viral infections altogether. This will be prospectively studied as part of the HELPcB cohort in the next coming years.

## Supplementary Information

Below is the link to the electronic supplementary material.

Supplementary file1 (DOCX 35 KB)
