# Supplementary material for: Longitudinal changes in telomere length in PCB-exposed individuals: interaction with CMV infection
Source: Arch Toxicol. 2021 Mar 19;95(4):1517–20. doi: 10.1007/s00204-021-03019-x (PMC8032634; doi:10.1007/s00204-021-03019-x)
Supplement: Supplementary file 1 — Supplementary file1 (DOCX 35 KB) [file 204_2021_3019_MOESM1_ESM.docx]

**Supplementary information to: Longitudinal changes in telomere length in PCB exposed individuals: interaction with CMV infection**

Fabian Beier^1,+^, Andre Esser^2,+^, Lucia Vankann^1^ , Anne Abels^1^, Thomas Schettgen^2^, Thomas Kraus^2^, Tim H. Brümmendorf^1^ and Patrick Ziegler^2*^

1. Department of Hematology, Oncology, Hemostaseology, and Stem Cell Transplantation, Medical Faculty, RWTH Aachen University, Aachen, Germany.

2. Institute for Occupational, Social and Environmental Medicine, RWTH Aachen University, Pauwelsstraße 30, 52074 Aachen, North Rhine Westphalia, Germany.

+ Both authors contributed equally

* correspondence to:[pziegler@ukaachen.de](mailto:pziegler@ukaachen.de)

**Patients and methods**

**Participants and sample aquisition**

This analysis is part of the German HELPcB surveillance program (Health Effects in high Level exposure to PCB) for occupationally exposed workers and their relatives (Kraus et al. 2012). The surveillance program was approved by the local ethics committee of the Medical Faculty of the RWTH Aachen University, Germany (EK 176/11). TL was assessed at two time points (2011; 207 participants) with a six-year follow-up (2017; 95 participants). CMV IgG levels were measured in house at the *l*aboratory diagnostics center (2015;119 participants). PCBs were analyzed in plasma via human biomonitoring as described (Schettgen et al. 2012).

**Flow Cytometry based fluorescence in situ Hybridization** (**Flow-FISH).**

Flow-FISH was carried out according to previously described protocols (Bouillon et al. 2018) (Ferreira et al. 2020). Briefly, samples were analyzed in triplicates with and without Alexa-488 labelled C-rich telomere probe (Eurogentech, Germany). Cow thymocytes with a previously determined telomere length were used both as an internal control as well as a standard to translate telomere fluorescence into kb. Cow thymocytes, granulocytes and lymphocytes were identified based on forward scatter properties and LDS 751 fluorescence. TL was determined in absolute values and in relation to the respective calculated age expressed as age adjusted telomere length (ΔTel). Blood of 265 unexposed individuals were used for age-adaption as described previously (Werner et al. 2015).

# **Statistics**

Kolmogorov-Smirnow-Lilliefors test and Shapiro Wilk test were used to determine Gaussian distribution of TL data from 2011 and 2017. In addition, histograms and QQ plots were visually inspected. For TL data from 2017 the Shapiro Wilk test showed a non-normal distribution, but histograms and QQ plots confirmed overall distribution close enough to the Gaussian model which led us to use parametric methods for further analysis (Schmider et al. 2010). First an ANCOVA with repeated measurements was performed. The age-adjusted TL was used as factor levels in 2011 and 2017 and the development for CMV positive and negative subjects was considered separately. A dichotomous dummy variable with 1 for PCB concentrations above the median and 0 for values below the median was used as predictor. The covariates cumulative pack-years for smokers and ex-smokers, as well as the daily alcohol consumption in g/d, as determined by questionnaires, were included.

To represent the tripple nested structure of the data (repeated measurement, CMV pos./neg, PCB concentration), a mixed model analysis was developed. In addition, a term for the interaction between PCB load and CMV+/- was added to the equation.

For the development of the model, zero models were formed to investigate the respective variance elucidation by grouping factors (Bliese 2016). Intra-class correlation (ICC), Akaike- information-criterion (AIC) and Bayesia-information-criterion (BIC) were used as criteria for the quality of the respective model. A double grouping factor in the random term for CMV+/- and the respective identification (ID) number of the participant at the respective time point of measurement was the best fitting model. The following variables were included in the equitation: PCB concentration, CMV pos./neg., cumulative pack years of smokers and ex-smokers as well as the interaction of PCB concentration in 2011 with the CMV serostate. Since the amount of alcohol consumed per day in g/d did not have a significant influence in ANCOVA, it was omitted from the mixed model analysis. All calculations were conducted with SPSS 25 (IBM 2017) and R 3.3.3 (R-Core-Team 2017).

# Supplemental Table 1: Results of ANCOVA

| **Tests of Between-Subjects Effects PCB 28** | | | | | | |
| --- | --- | --- | --- | --- | --- | --- |
| Source | Type III Sum of Squares | df | Mean Square | F | Sig. | Partial Eta Squared |
| Intercept | 68,968 | 1 | 68,968 | 49,645 | 0,000 | 0,398 |
| cumulative pack years 2011 | 9,529 | 1 | 9,529 | 6,860 | 0,011 | 0,084 |
| daily alcohol intake | 2,019 | 1 | 2,019 | 1,453 | 0,232 | 0,019 |
| Median_PCB28_2011 | 11,614 | 1 | 11,614 | 8,360 | 0,005 | 0,100 |
| CMV pos/neg | 0,778 | 1 | 0,778 | 0,560 | 0,457 | 0,007 |
| Median_PCB28_2011 * CMV | 1,065 | 1 | 1,065 | 0,767 | 0,384 | 0,010 |
| a. Computed using alpha = ,05 | | | | | | |
| **Tests of Between-Subjects Effects PCB 52** | | | | | | |
| Source | Type III Sum of Squares | df | Mean Square | F | Sig. | Partial Eta Squared |
| Intercept | 80,087 | 1 | 80,087 | 65,264 | 0,000 | 0,465 |
| cumulative pack years 2011 | 9,439 | 1 | 9,439 | 7,692 | 0,007 | 0,093 |
| daily alcohol intake | 2,162 | 1 | 2,162 | 1,762 | 0,188 | 0,023 |
| Median_PCB52_11 | 25,296 | 1 | 25,296 | 20,614 | 0,000 | 0,216 |
| CMV | 0,070 | 1 | 0,070 | 0,057 | 0,812 | 0,001 |
| Median_PCB52_11 * CMV | 0,012 | 1 | 0,012 | 0,010 | 0,921 | 0,000 |
| a. Computed using alpha = ,05 | | | | | | |
| **Tests of Between-Subjects Effects PCB 101** | | | | | | |
| Source | Type III Sum of Squares | df | Mean Square | F | Sig. | Partial Eta Squared |
| Intercept | 69,541 | 1 | 69,541 | 47,303 | 0,000 | 0,387 |
| cumulative pack years 2011 | 9,967 | 1 | 9,967 | 6,780 | 0,011 | 0,083 |
| daily alcohol intake | 2,727 | 1 | 2,727 | 1,855 | 0,177 | 0,024 |
| Median_PCB101_11 | 6,950 | 1 | 6,950 | 4,727 | 0,033 | 0,059 |
| CMV | 0,176 | 1 | 0,176 | 0,120 | 0,730 | 0,002 |
| Median_PCB101_11 * CMV | 0,070 | 1 | 0,070 | 0,047 | 0,828 | 0,001 |
| a. Computed using alpha = ,05 | | | | | | |
| **Tests of Between-Subjects Effects non-dioxin like PCB-Sum** | | | | | | |
| Source | Type III Sum of Squares | df | Mean Square | F | Sig. | Partial Eta Squared |
| Intercept | 68,968 | 1 | 68,968 | 49,645 | 0,000 | 0,398 |
| cumulative pack years 2011 | 9,529 | 1 | 9,529 | 6,860 | 0,011 | 0,084 |
| daily alcohol intake | 2,019 | 1 | 2,019 | 1,453 | 0,232 | 0,019 |
| Median_Sum_11 | 11,614 | 1 | 11,614 | 8,360 | 0,005 | 0,100 |
| CMV | 0,778 | 1 | 0,778 | 0,560 | 0,457 | 0,007 |
| Median_Sum_11 * CMV | 1,065 | 1 | 1,065 | 0,767 | 0,384 | 0,010 |
| a. Computed using alpha = ,05 | | | | | | |

Supplemental Table 2: Results of linear mixed effects model analysis

P-values obtained by mode-anova, sequential square sums used

| **model** | **linear mixed effects model** | **B** | **SE(B)** |  | **t-value** | **p-value** |
| --- | --- | --- | --- | --- | --- | --- |
| **PCB 28** | intercept | -0.653 | 0.152 |  | -4.287 | <0.001 |
|  | PCB28 | -0.103 | 0.069 |  | -1.487 | 0.037 |
|  | cum. pyears | 0.012 | 0.004 |  | 2.780 | 0.007 |
|  | CMV pos./neg. | 0.208 | 0.213 |  | 0.978 | 0.562 |
|  | IA PCB28:CMVpos/neg | -0.731 | 0.257 |  | -2.840 | 0.006 |
| **PCB52** | intercept | -0.660 | 0.153 |  | -4.326 | <0.001 |
|  | PCB52 | -1.004 | 1.294 |  | -0.776 | 0.090 |
|  | cum. pyears | 0.011 | 0.004 |  | 2.509 | 0.007 |
|  | CMV pos./neg. | 0.187 | 0.206 |  | 0.908 | 0.634 |
|  | IA PCB52:CMVpos/neg | -0.758 | 0.266 |  | -2.854 | 0.006 |
| **PCB101** | intercept | -0.665 | 0.157 |  | -4.234 | <0.001 |
|  | PCB101 | -0.742 | 0.656 |  | -1.131 | 0.087 |
|  | cum. pyears | 0.012 | 0.004 |  | 2.888 | 0.009 |
|  | CMV pos./neg. | 0.162 | 0.226 |  | 0.716 | 0.576 |
|  | IA PCB101:CMVpos/neg | -0.548 | 0.265 |  | -2.065 | 0.042 |
| **ndlPCB_sum** | intercept | -0.651 | 0.153 |  | -4.266 | <0.001 |
|  | ndlPCB_Sum | -0.095 | 0.063 |  | -1.504 | 0.031 |
|  | cum. pyears | 0.012 | 0.004 |  | 2.772 | 0.007 |
|  | CMV pos./neg. | 0.207 | 0.213 |  | 0.973 | 0.572 |
|  | IA ndlPCB_Sum :CMVpos/neg | -0.725 | 0.258 |  | -2.808 | 0.006 |

**References**

Bliese P (2016) multilevel: Multilevel Functions. R package version 2.6 edn. R Foundation for Statistical Computing

Bouillon AS, Ventura Ferreira MS, Awad SA, et al. (2018) Telomere shortening correlates with leukemic stem cell burden at diagnosis of chronic myeloid leukemia. Blood advances 2(13):1572-1579 doi:10.1182/bloodadvances.2018017772

Ferreira MSV, Kirschner M, Halfmeyer I, et al. (2020) Comparison of flow-FISH and MM-qPCR telomere length assessment techniques for the screening of telomeropathies. Annals of the New York Academy of Sciences 1466(1):93-103 doi:10.1111/nyas.14248

IBM (2017) SPSS Statistics for Windows vol Version 25, Armonk, N.Y.

Kraus T, Gube M, Lang J, et al. (2012) Surveillance program for former PCB-exposed workers of a transformer and capacitor recycling company, family members, employees of surrounding companies, and area residents--executive summary. Journal of toxicology and environmental health Part A 75(19-20):1241-7 doi:10.1080/15287394.2012.709377

R-Core-Team (2017) R: A Language and Environment for Statistical Computing. 3.3.3 (2017-03-06) edn. R Foundation for Statistical Computing, Vienna, Austria

Schettgen T, Gube M, Esser A, Alt A, Kraus T (2012) Plasma polychlorinated biphenyls (PCB) levels of workers in a transformer recycling company, their family members, and employees of surrounding companies. Journal of toxicology and environmental health Part A 75(8-10):414-22 doi:10.1080/15287394.2012.674905

Schmider E, Ziegler M, Danay E, Beyer L, Buehner M (2010) Is It Really Robust? Reinvestigating the Robustness of ANOVA Against Violations of the Normal Distribution Assumption. Methodology-European Journal of Research Methods for the Behavioral and Social Sciences 6(4):147-151 doi:10.1027/1614-2241/a000016

Werner B, Beier F, Hummel S, et al. (2015) Reconstructing the in vivo dynamics of hematopoietic stem cells from telomere length distributions. eLife 4 doi:10.7554/eLife.08687
